# Nanomaterials-based biosensor and their applications: A review

**DOI:** 10.1016/j.heliyon.2023.e19929

**Published:** 2023-09-07

**Authors:** Sumit Malik, Joginder Singh, Rohit Goyat, Yajvinder Saharan, Vivek Chaudhry, Ahmad Umar, Ahmed A. Ibrahim, Sheikh Akbar, Sadia Ameen, Sotirios Baskoutas

**Affiliations:** a Department of Chemistry, Maharishi Markandeshwar (Deemed to be University), Mullana, Ambala, 133203, Haryana, India; b Department of Chemistry, Faculty of Science and Arts, and Promising Centre for Sensors and Electronic Devices (PCSED)Najran University, Najran, 11001, Kingdom of Saudi Arabia; c Department of Materials Science and Engineering, The Ohio State University, Columbus, OH, 43210, USA; d Advanced Materials and Devices Laboratory, Department of Bio-Convergence Science, Advanced Science Campus, Jeonbuk National University, 56212, Jeonju, Republic of Korea; e Department of Materials Science, University of Patras, 26500, Patras, Greece; f Department of Materials Science and Engineering, The Ohio State University, Columbus, OH, 43210, USA

**Keywords:** Nanowires, Nanorods, Carbon nanotubes, Quantum dots, Dendrimers

## Abstract

A sensor can be called ideal or perfect if it is enriched with certain characteristics viz., superior detections range, high sensitivity, selectivity, resolution, reproducibility, repeatability, and response time with good flow. Recently, biosensors made of nanoparticles (NPs) have gained very high popularity due to their excellent applications in nearly all the fields of science and technology. The use of NPs in the biosensor is usually done to fill the gap between the converter and the bioreceptor, which is at the nanoscale. Simultaneously the uses of NPs and electrochemical techniques have led to the emergence of biosensors with high sensitivity and decomposition power. This review summarizes the development of biosensors made of NPssuch as noble metal NPs and metal oxide NPs, nanowires (NWs), nanorods (NRs), carbon nanotubes (CNTs), quantum dots (QDs), and dendrimers and their recent advancement in biosensing technology with the expansion of nanotechnology.

## 1 Introduction

**Fig. 1:**
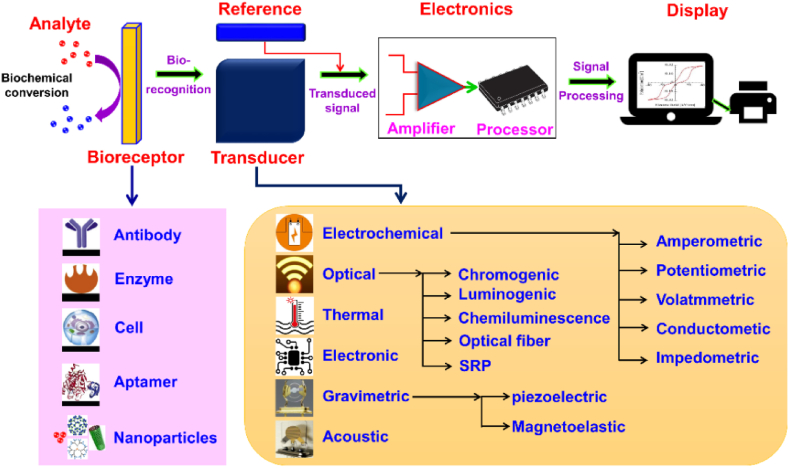
Schematic diagram of biosensor consisting of bioreceptor, transducer, and amplifier. Reproduced with permission from Ref. [3], with the permission of the Creative Commons Attribution 4.0 International License (http://creativecommons.org/licenses/by/4.0/). Copyright 2021, MDPI.

Diseases if detected in early stages can increase the chance of successful treatments and survival, hence it is the need of hour to develop a device which can detect or sense the trouble causing organic/inorganic biomolecule in the living organism [1]. The American biochemist L.L Clark was the first person to invent the biosensor in year 1956. He used this biosensor to detect percentage of oxygen in the blood and the electrode he used in this sensor was named as the Clark electrode or oxygen electrode [2]. The term biosensor was first used by Cammann in 1977 [1] and it can be defined as analytical device which is designed by the combination of bioreceptor (cell, enzyme, antibody, DNA etc.), transducer (translate the observed signal into a useful output) and an amplifier (amplifies and processes the final signal) as depicted in Fig. 1. Further, a substance of curiosity, which needs to be detected, is called an analyte.

The development in the area of biosensors is divided into mainly five generations:–In the first generation, the biosensors measure two different things, first the composition of the analyte and second the bioreceptor reactions product, which finally produces the signal as a response. Leland Charles Clark Jr. was the first one to work on this type of biosensor [2]. In 1962, Clark further employed an amperometric enzyme electrode for glucose detection [4]. Later, in 1967, Updike and Hicks, modified the Clark's work and prepared the first enzyme electrode [5]. Further in the year 1969, Guilbault and Montalvo, designed a potentiometric electrode sensor for sensing urea [6]. Similarly in 1973, Guilbault and Lubrano detected hydrogen peroxide using lactate/glucose enzyme-based sensor [7]. The design and development of thermistors was carried out by Klaus Mosbach group in 1974 for detecting temperature variations [8]. In 1975, Lubbers and Opitz prepared an optical biosensor for the sensing of alcohol [9]. Fig. 1 shows the schematic diagram for the fundamental components of a biosensor, highlighting the critical elements of the bioreceptor, transducer, and amplifier. This visual representation provides a clear understanding of the essential building blocks that collaborate to enable accurate and sensitive biosensing capabilities.

**Fig. 2:**
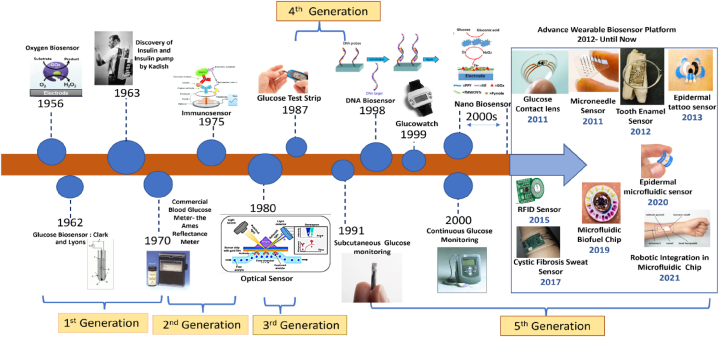
The different milestones achieved in the field of biosensors. Reproduced with permission from Ref. [12], with the permission of the Creative Commons Attribution 4.0 International License (http://creativecommons.org/licenses/by/4.0/). Copyright 2022, MDPI.

In the 2nd generation biosensors, the analytical efficiency of the biosensor was enhanced by the addition of auxiliary enzymes and co-reactants [10]. These sensors were named as mediator amperometric biosensors. In the third to fifth generation, the bio-receptors become an essential part of the sensing element. The direct interface was created between the bioreceptor (enzymes) and electrode via the transfer of electrons, without involving any intermediate. The main advantages of these generation sensors were their low cost and repeatability with high sensitivity [11]. The different milestones achieved in the field of biosensors are shown in Fig. 2. In Fig. 2, a visual representation illustrates the significant milestones that have been accomplished in the dynamic field of biosensors. This comprehensive overview highlights the key advancements and breakthroughs that have shaped the evolution of biosensing technologies, highlighting the remarkable progress achieved over time.

### 1.1 Characteristics and classification of biosensors

The designed and prepared biosensor prototype must possess the following characteristics to the better end so that the desired results can be achieved for the upliftment and betterment of the health of the society.

**Fig. 3:**
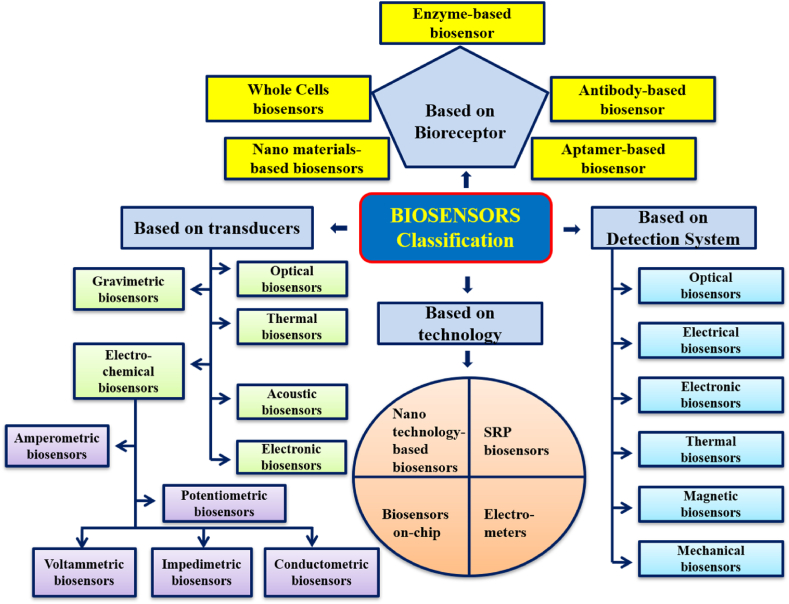
Classification of biosensors based on detection system, transducer, technology and bio-receptors.
(i) Biosensor based on the type of bio-receptors used for preparing the device
(ii) Biosensor based on the type of transducer used for making the device
(iii) Biosensor based on the type of technology used for designing the device
(iv) Biosensor based on the type of detection system used.

(i) **Selectivity:** Before designing any biosensor, the main thought in the mind of the designer is to think about the selectivity of the biosensor so that it can detect the desired analyte from sample containing different or nearly similar analytes/contaminants [3]. Hence selectivity is the most important feature of the biosensor.
(ii) **Reproducibility:** The ability of the biosensor to reproduce the same results again and again for duplicated experiments is an immense matter of concern [13]. The biosensors with high reproducibility quality are really in high demand at present. Along with reproducibility, the results obtained should be of high accuracy and precision and altogether these properties of the biosensor make it the more dependable one.
(iii) **Stability:** The stability of the biosensor is quite crucial factor which counts for the commercial successes of the biosensor. The biosensor does lose their strength of signals with age, hence this factor needs more consideration and attention [3]. Further the ageing or instability get accelerated with rise in temperature or in other words ageing in directly temperature dependent.
(iv) **Sensitivity and Linearity:** The biosensors are rated high only if they possess high sensitivity. In today's world especially in air, water and soil pollutant detection, the requirement is a ppm level whereas in medical field it goes from nanograms per milliliter to femtograms per milliliter [13]. Further linearity of the device highlights the accuracy of the given response for a given set of measurements versus different concentration of an analyte. Fig. 3 illustrates a comprehensive classification of biosensors, categorized based on their detection system, transducer technology, and choice of bio-receptors. This classification offers insights into the diverse range of biosensing approaches, aiding researchers in selecting the most suitable biosensor design for their specific applications. Regarding the classification of biosensors, the different criterion and factors can be employed. Here in this review article the classification has been chalked out based on four main criterions as shown in Fig. 3 and are detailed as below:

Further, this article will be focused on only the nano material-based biosensors as they are finding more importance and have wider applications.

## 2 Advancement of nanotechnology and NPs-based biosensors

To meet up the heavy demand of biosensors nearly in all fields of science and technology, scientist community have been forced to explore new materials at nanoscale level which could be employed in the sensor technology to achieve good results. Opioids, widely employed as potent analgesics for pain management, bear the dual nature of therapeutic benefits and potential risks. Instances of overdose and the risk of developing addictive behaviors underscore the need for vigilant monitoring. The surge in illicit drug consumption and misuse on a global scale necessitates precise and efficient detection methodologies across confiscated samples, biological matrices, and environmental effluents. In this context, the integration of advanced nanostructures into biosensing platforms offers a promising avenue for opioid detection, enabling rapid and accurate identification. A recent scholarly contribution by Saman Sargazi and colleagues delves into the realm of nanobiosensors tailored for opioids, offering a comprehensive analysis of the burgeoning field [14]. This review meticulously navigates the landscape of nanomaterials, exemplifying their application as biosensing tools targeting opioids. The focus extends to the molecular entities under scrutiny and the associated limits of detection, collectively shaping the precision and scope of these nanobiosensor systems.

**Fig. 4:**
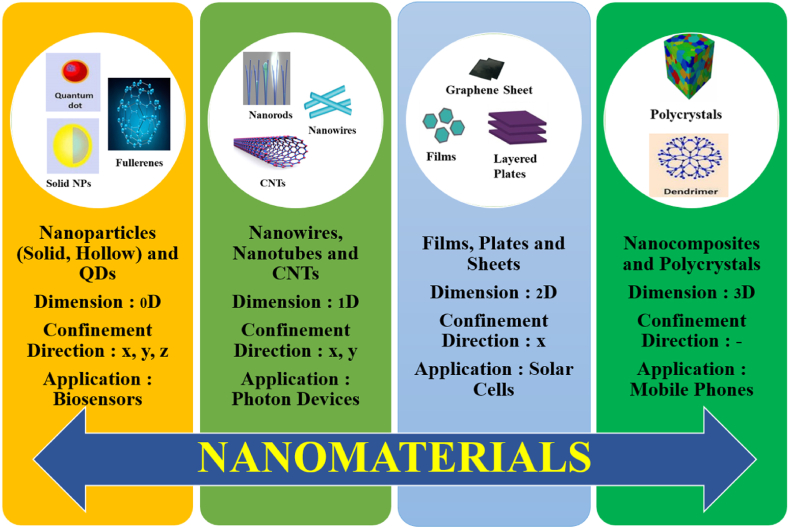
Different types of NMs with different dimensions utilized in designing biosensors.

In last two to three decades, nanotechnology has shown great advancement in its development and applications [15]. Numerous NPs, nanomaterials have been synthesized, designed and are utilized in enhancing the overall performances of the biosensors [16]. In Fig. 4, a depiction showcases the diverse array of nanomaterials (NMs) employed in the design of biosensors, highlighting their distinct dimensions and characteristics. This visual representation offers insights into the various types of nanomaterials utilized, underlining their significant role in enhancing the performance and sensitivity of biosensing platforms.

**Fig. 5:**
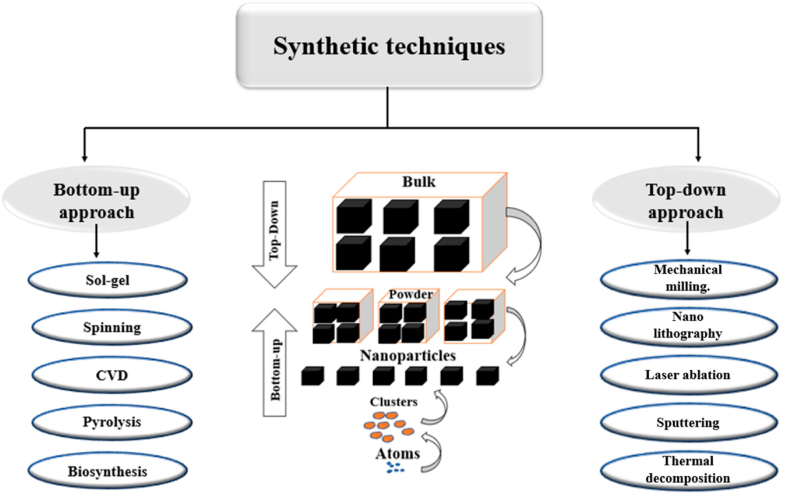
Top-down and bottom-up methodologies for preparing nanomaterials. Reproduced with permission from Ref. [18], with the permission of the Creative Commons Attribution 4.0 International License (http://creativecommons.org/licenses/by/4.0/). Copyright 2022, MDPI.

Several methods and technologies [17] have been designed and opted for preparing nanomaterials, including a “top-down” (bulk materials are reconstituted to form nanoscale materials) and a “bottom-up” methodologies (nano-scale materials are assembled at the molecular level) as shown in Fig. 5 [18]. Fig. 5 exhibits the schematic for the top-down and bottom-up methodologies employed for the preparation of nanomaterials. This illustration provides a clear overview of these distinct approaches, showcasing how the nanomaterials are synthesized from macroscopic to nanoscale dimensions (top down) or prepare from individual components to form larger structures (bottom up). The bottom-up approach encompasses a multitude of techniques, including sol-gel, spinning, chemical vapor deposition (CVD), pyrolysis, biosynthesis, hydrothermal synthesis, and more. Similarly, the top-down approach comprises a diverse array of methods such as mechanical milling, lithography, laser ablation, sputtering, thermal decomposition, and others. This comprehensive range of techniques underscores the versatility and complexity of both bottom-up and top-down methodologies in nanoparticle synthesis. The intricate investigation and systematic development of metal oxide-based nanomaterials, such as nanowires (NWs), nanorods (NRs), carbon nanotubes (CNTs), quantum dots (QDs), and nanocomposite dendrimers, have the potential to revolutionize the landscape of biosensor technology. By delving into the design and synthesis of these nanomaterials, it becomes possible to unlock new dimensions in enhancing the detection capabilities of biosensors. These nanomaterials offer a remarkable platform for manipulation and customization, allowing researchers to finely tune their properties to precisely match the requirements of diverse biosensing applications. This level of precision engineering not only empowers biosensors to achieve higher sensitivities but also opens avenues to controlling their selectivity and overall performance.

### 2.1 Metal oxide-based biosensors

Metal oxide-based nanomaterials, known for their unique physicochemical properties at the nanoscale, hold the promise of elevating the sensitivity and responsiveness of biosensors. Nanowires and nanorods provide a one-dimensional architecture that can facilitate efficient charge transfer and signal transduction. In addition, such nanomaterials offer exceptional mechanical, electrical, and thermal properties, and all of which can be harnessed to enhance biosensor functionality. Quantum dots, with their tunable optical properties, enable precise signal amplification and multiplexing, adding a new layer of sophistication to biosensor designs. Moreover, the incorporation of nanocomposite dendrimers offers a versatile platform for functionalizing biosensor surfaces, enhancing binding interactions, and improving stability, thereby contributing to the overall robustness of biosensor performance.

As the realms of nanotechnology and biosensor development converge, the potential for groundbreaking advancements in detection capabilities becomes evident. The careful manipulation and incorporation of these nanomaterials into biosensor designs pave the way for unparalleled sensitivity, specificity, and efficiency in detecting target analytes. By harnessing the inherent advantages of these nanoscale structures, researchers can push the boundaries of biosensor technology and redefine its role in various fields, ranging from healthcare diagnostics to environmental monitoring and beyond.

In the past two decades, oxides of copper (CuO), nickel (NiO), iron (Fe_2_O_3_), cobalt (Co_3_O_4_), manganese (MnO_2_), zinc (ZnO), tin (SnO_2_), titanium (TiO_2_) and cadmium (CdO) etc. have been extensively engaged in a variety of fields virtue of their extensive range of electrical, chemical and physical properties. Among the above-mentioned metal oxides, oxides of zinc, copper, iron and manganese are adopted as the best magnetic nanomaterials showing high electron movement rate hence utilized in designing electrochemical biosensors [19].

#### 2.1.1 Zinc oxide-based biosensor

**Fig. 6:**
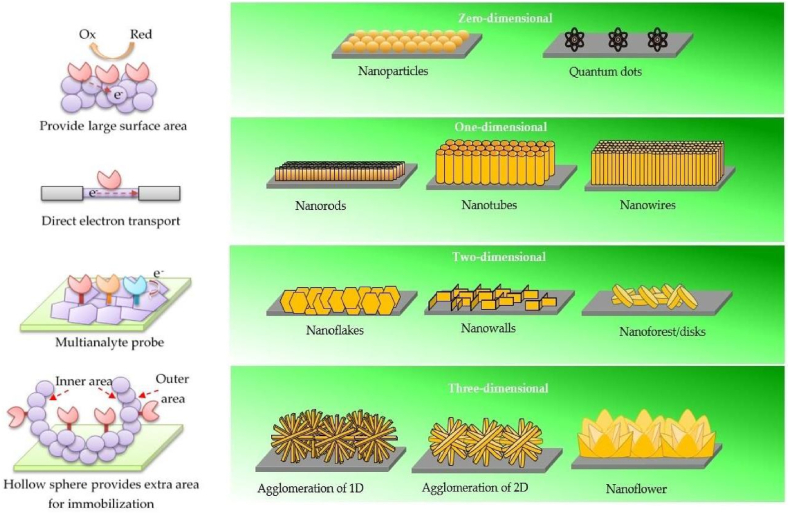
The four different shapes of ZnO nanostructures with their characteristics. Reproduced with permission from Ref. [22], with the permission of the Creative Commons Attribution 4.0 International License (http://creativecommons.org/licenses/by/4.0/). Copyright 2019, MDPI.

ZnO has been identified an excellent candidate for designing a biosensor virtue of its high isoelectric point (IEP), cost effectiveness, eco-friendly nature, and chemical stability. A high value of IEP allows enhanced absorption process of the analytes such as enzymes, DNA, and proteins by electrostatic interactions. Further its properties viz.; an n-type semiconductor with broad band gap (3.37eV), high exciton binding energy (6.0 meV) and good electron mobility makes it more promising material in fabrications of biosensors [20]. The broad band gap helps ZnO to sustain large electric ﬁelds, which allow a high breakdown voltage and stable semiconductor in the visible region [21]. Along with this, to enhance its application to wider range ZnO NPs have four different dimensions starting from zero dimension (0-D) to 3-D. The illustration in Fig. 6 highlights the diverse dimensions of ZnO nanostructures, each offering distinct advantages for biosensor applications. These advantages play a pivotal role in enhancing the overall performance and functionality of biosensors, catering to specific detection needs.

**0-D Nanostructures (Zero-Dimensional)**: Zero-dimensional nanostructures present a vast surface area. This expansive surface offers ample room for immobilizing biomolecules, enabling efficient interactions between target analytes and sensing elements. The large surface-to-volume ratio enhances sensitivity, making these structures well-suited for ultra-sensitive biosensing applications.

**1-D Nanostructures (One-Dimensional)**: One-dimensional nanostructures provide stable and direct electron transport pathways. The elongated structure facilitates efficient charge transfer, leading to enhanced signal transduction. This stability in electron transport results in improved sensor response and accuracy, making 1-D nanostructures an ideal choice for robust biosensor designs.

**2-D Nanostructures (Two-Dimensional)**: Two-dimensional nanostructures offer specific planes for immobilization processes. These immobilization planes enable the simultaneous detection of multiple analytes, making them highly valuable for multi-analyte biosensing. The versatility of 2-D nanostructures in accommodating various sensing elements enhances the sensor's capability to target and distinguish different analytes in complex samples.

**3-D Nanostructures (Three-Dimensional)**: Three-dimensional nanostructures encompass both outer and inner surfaces, providing additional sites for immobilization. This extra surface area facilitates the attachment of a higher number of biomolecules, thereby improving the sensitivity and binding efficiency of the biosensor. The increased surface area of 3-D nanostructures enhances the sensor's ability to capture and detect trace amounts of target analytes.

Collectively, the distinct advantages associated with each dimension of ZnO nanostructures contribute to the advancement of biosensor performance. By harnessing these advantages, biosensor designers can tailor their platforms to achieve specific detection goals, enabling applications ranging from medical diagnostics to environmental monitoring and beyond.

ZnO has been used extensively used to sense various compounds such as glucose, ascorbic acid, cholesterol, uric acid, and cancer cells etc. In 2014, Tashkhourian et al. fabricated an effective naproxen electrochemical sensor by using carbon paste electrode modified with ZnO NPs and multiwalled carbon nanotubes. It was detected by square wave voltammetry with a linear concentration range of 1.0 × 10^−6^ M to 2.0 × 10^−4^ M and the detection limit was 2.3 × 10^−7^ M [23]. In year 2015, Roy et al. prepared a novel Ag–ZnO bimetallic, graphene oxide coated polymer-based sensor for the detection, of *E. coli* bacteria. It detected concentrated in the range of 10^5^ CFU mL^−1^ and detected as low as 10 CFUmL^−1^ [24]. In the same year, Bashami et al. fabricated the ZnO coated carbon electrode for the sensitive detection of *para*-nitrophenol. It was detected over a linear concentration range from 2.1 μM to 6.3 μM and the lower detection limit was 0.02 μM [25]. Further in the year 2016, Fang et al. developed 3-D ZnO sensors using trisodium citrate-assisted solution phase method for sensing glucose. It detected glucose in linear range from 1 to 20 mM and the lower detection limit was 0.02 mM [26].

**Fig. 7:**
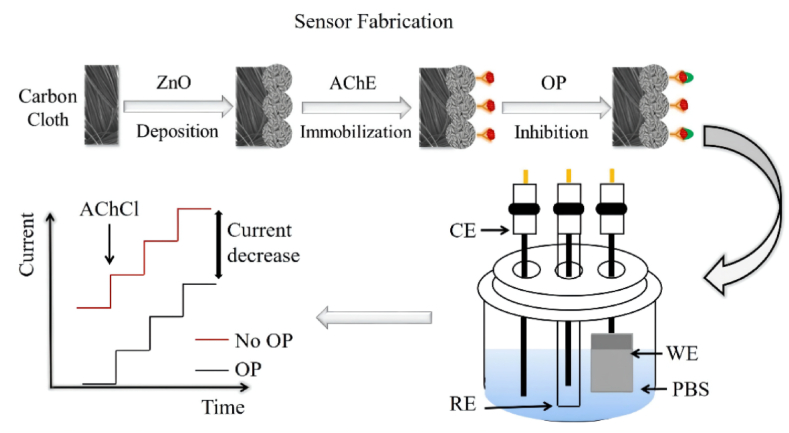
ZnO based biosensor for pesticide detection. Reproduced with permission from Ref. [27], with the permission of the Creative Commons Attribution 4.0 International License (http://creativecommons.org/licenses/by/4.0/). Copyright 2022, MDPI.

The development of an affordable, sensitive, and portable biosensor for detecting pesticides holds significance in various applications, including food packaging, agriculture, and environmental monitoring. In the year 2022, Fallatah et al. prepared a zinc oxide (ZnO) nanostructure-based biosensor that can be formed on a flexible porous surface for the detection of pesticides, as shown in Fig. 7. The biosensors were constructed by immobilizing the acetylcholinesterase (AChE) enzyme on ZnO, which was directly grown on the flexible substrates. Notably, the ZnO biosensors developed on carbon cloth exhibited superior performance characteristics, including a detection limit for OP ranging from 0.5 nM to 5 μM, heightened sensitivity, and enhanced stability [27].

#### 2.1.2 Copper oxide-based biosensor

The oxides of copper viz.; CuO and Cu_2_O are non-poisonous nanomaterials and that could be easily fabricated in the abundant amount at quite effective low cost. Further their synthesis can be tailored to obtain the NPs of high crystalline nature with required size and shape that can be employed in fabricating biosensors of severe demand. Along with fabricating sensor devices, the oxides of copper being p-type semiconductor are also in high demand for making batteries, supercapacitors, photovoltaic cells and field emission devices etc. [28,29].

Hydrogen peroxide (H_2_O_2_) serves as a potent oxidant and bleaching agent with widespread applications across biomedicine, households, and industries. Additionally, H_2_O_2_ functions as a significant reactive oxygen species (ROS) implicated in various physiological and pathological processes. Its connection to a range of human diseases, including cardiovascular disorders, diabetes, neurodegenerative conditions such as Parkinson's disease, Alzheimer's disease, Huntington's disease, as well as metabolic disorders and cancers, underscores its critical role. Consequently, the accurate detection of H_2_O_2_ holds paramount importance for both academic research and industrial applications. Addressing this need, the development of H_2_O_2_ sensors that are cost-effective, rapid, sensitive, and selective is imperative. In current times, a plethora of sensor platforms has emerged to detect hydrogen peroxide.

Ping et al. fabricated carbon ionic liquid electrode with copper oxide NPs for sensing H_2_O_2_. It was detected over a continuous range from 1.0 μM to 2.5 mM and the lower detection limit was 0.5 μM [30]. Dhara et al. prepared a biosensor by decorating reduced reduce graphene oxide with palladium-copper oxide NPs for the detection of glucose, with the linear concentration range from 6 μM to 22 mM and the lower limit of detection was 30 nM [31]. Z. Monsef Khoshhesab developed electrochemical sensor based on CuO-graphene nanocomposite for the simultaneous detection of acetaminophen, ascorbic acid and caffeine. It was detected over a linear range from 0.025 to 5.3 μmol L^−1^ and the limit of detection were 0.008, 0.011 and 0.010 μmol L^−1^ respectively [32]. Similarly, Zhang et al. synthesized CuO NPs decorated with carbon spheres (CuONPs-CSs) for the electrochemical determination of glucose with a linear concentration range from 5.0 × 10^−7^ to 2.3 × 10^−3^ M, the detection limit was 0.1 μM and the high sensitivity of 2981 μA mM^−1^ cm^−2^ [33]. Table 1 highlights the different cuprous/cupric oxide NPs based electrochemical biosensors used in recent years.

**Table 1:** The different cuprous/cupric oxide NPs based electrochemical biosensors.

S.No.	MOs Nanomaterials	Analyte	Sensing Method	Linear Range	Detection Limit	Ref.
1.	CuO/g-C_3_N_4_ nanocomposites	Dopamine	Electrochemical	2 × 10^−9^–7.11 × 10^−5^ molL^−1^	1 × 10^−10^ molL^−1^	[34]
2.	CuO/GO	Glucose	Electrochemical	0.0028––2.03 mM	0.69 μM	[35]
3.	Cu_x_O/ERGO	Dopamine	Electrochemical	0.1––400 μM	12 nM	[36]
4.	CuO NPs on Carbon ceramic electrode	Tyrosine	Amperometry	2––1350 μM	160 nM	[37]
5.	CuO-rGO	Glucose	Amperometry	0.0004––12 mM	0.1 μM	[38]
6.	Cu_2_O–TiNTs	Eugenol	Cyclic Voltammetry	4.6––130 μM	1.3 μM	[39]
7.	Cu_2_O–rGO/GCE	H_2_O_2_	Amperometry	0.03––12.8 mM	21.7 μM	[40]
8.	CuO-Gr/CPE	Acetaminophen	DPV	0.025––5.3 μM	0.008 μM	[41]
9.	Cu_2_O-BSA NPs	Glucose	Cyclic Voltammetry	Up to 10 mM	0.4 μM	[42]
10.	CuO-Gr/CPE	Caffeine	DPV	0.025––0.3 μM	0.010 μM	[41]

**Fig. 8:**
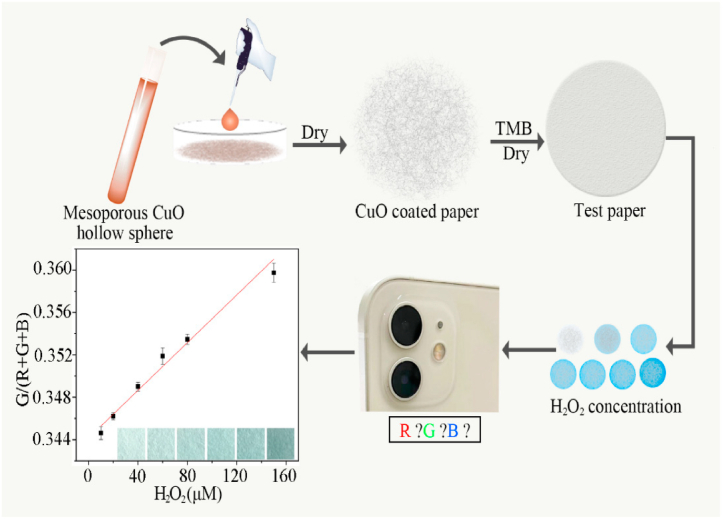
Schematic for the paper based colorimetric sensor using mesoporous copper oxide CuO hollow sphere for the detection of hydrogen peroxide. Reproduced with permission from Ref. [43], with the permission of the Creative Commons Attribution 4.0 International License (http://creativecommons.org/licenses/by/4.0/). Copyright 2021, MDPI.

Another illustrative example by Cheng et al. introduces a paper-based colorimetric sensor utilizing mesoporous copper oxide (CuO) hollow spheres for H_2_O_2_ detection. These mesoporous CuO hollow spheres exhibit noteworthy characteristics, including a substantial specific surface area (58.77 m^2^/g), appreciable pore volume (0.56 cm^3^/g), accessible mesopores (5.8 nm), a hollow morphology, and uniform diameter (∼100 nm). Importantly, they demonstrate excellent peroxidase-like activities, with K_m_ and V_max_ values of 120 mM and 1.396 × 10^−5^ M s^−1^, respectively (Fig. 8). Leveraging these properties, the mesoporous CuO hollow spheres are employed on low-cost, disposable filter paper test strips. The resultant paper-based sensor exhibits efficacy in detecting H_2_O_2_ across a range of 2.4–150 μM. This innovative approach presents a promising avenue for efficient and reliable H_2_O_2_ detection, offering significant benefits for various applications [43].

#### 2.1.3 Iron oxide-based biosensor

The oxides of iron Fe_2_O_3_ and Fe_3_O_4_ have been utilized extensively in fabricating different types of electrodes for designing numerous types of biosensors for detecting heavy metal ions, organic molecules etc. In year 2009, Kaushik et al. fabricated a urea sensor by coating glass plate with indium-tin oxide and then depositing thin film of Fe_3_O_4_NPs/chitosan. Urea was detected with a concentration range of 5–100 mg/dL and with limit of detection was 0.5 mg/dL [44]. In year 2015, Li et al. developed a nitrite sensor by using the Ag–Fe_3_O_4_–graphene oxide magnetic nanocomposite, with a linear range of 0.5 μM–0.72 mM and the lower limit of detection was 0.17 μM [45]. In 2016, Lee et al. used Fe_2_O_3_/graphene NPs for fabricating electrochemical sensor for detecting Zn^2+^, Cd^2+^ and Pb^2+^ metal ions. These were detected over a linear range of 1–100 μg L^−1^ for Zn^2+^, Cd^2+^, and Pb^2+^ and the lower limit of detection were 0.11 μg L^−1^, 0.08 μg L^−1^, and 0.07 μg L^−1^ [46]. Further Table 2 depicts the Iron oxide NPs based electrochemical biosensors employed recently in different fields.

**Table 2:** The various iron oxide NPs based electrochemical biosensors.

S.No.	MOs Nanomaterials	Analyte	Sensing Method	Linear Range	Detection Limit	Ref.
1.	Fe_2_O_3_ NPs	Uric Acid	Electrochemical	10––100 μM	2.5 nM	[47]
2.	Fe_3_O_4_/rGO nanocomposite	Dopamine	Amperometry	0.010––0.270 μM	5 nM	[48]
3.	Fe_3_O_4_/rGO nanocomposite	Ascorbic Acid	DPV	1––9 mM	0.42 μM	[49]
4.	Fe_3_O_4_ modified Carbon paste electrode	Tyrosine	DPV	0.4––270.0 μM	50 nM	[50]
5.	rGO/Fe_3_O_4_/Gelatin	Glucose	Cyclic Voltammetry	0.1––10 mM	0.024 μM	[51]
6.	Polypyrrole-chitosan-Iron oxide	Glucose	Electrochemical	1––16 mM	234 μM	[52]
7.	Ag@Fe_2_O_3_/SPCE	Nitrate	Amperometry	0––1000 μM	30 μM	[53]
8.	Fe_3_O_4_NPs-CB/GCE	Bisphenol A	DPV	0.1 nM–50 μM	0.031 nM	[54]
9.	Fe_2_O_3_/GCE	Pyrocatechol	Chronoamperometry	7.9––130 μM		[55]
10.	PEG- Fe_3_O_4_/GE	l- Dopa	DPV	0.05––10 μM	9.5 nM	[56]

Iron oxide (Fe_2_O_3_) has emerged as a versatile transition metal oxide renowned for its affordability, abundance, favorable biocompatibility, and impressive electrochemical attributes, rendering it a material of significant interest across various domains. Among its myriad applications, α-Fe_2_O_3_ nanoparticles (α-Fe_2_O_3_ NPs) have garnered substantial attention as an exceptional modifying agent. This is attributed to the inherent capability of iron oxides to undergo in situ electrochemical reduction or oxidation, owing to their variable valence state, thereby inducing heterogeneous redox reactions pertinent to the target analyte. Notably, investigations have showcased the influential role of nanostructured α- Fe_2_O_3_ morphologies on optical, magnetic, photocatalytic, and electrochemical properties. Intriguingly, the impact of morphology on electrochemical sensing, particularly concerning small biomolecules, remains an area warranting exploration.

**Fig. 9:**
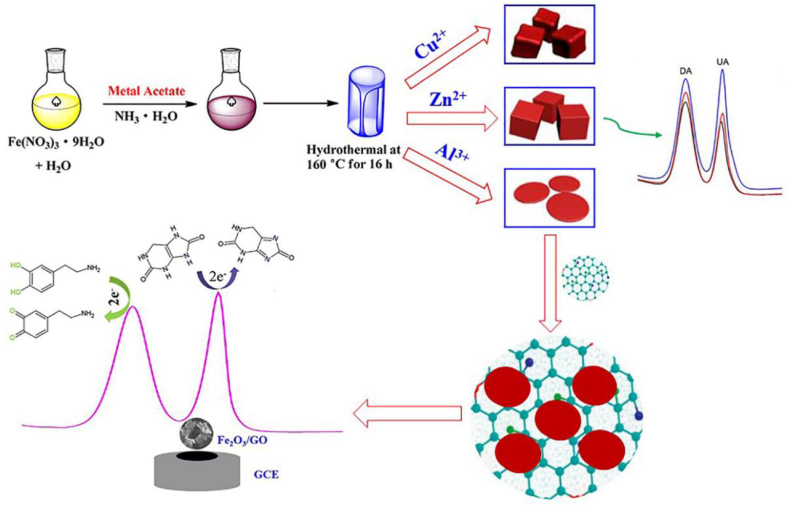
Fe_2_O_3_/GO/GCE based electrochemical sensor for the detection of dopamine and uric acid. Reproduced with permission from Ref. [58], with the permission of the Creative Commons Attribution 4.0 International License (http://creativecommons.org/licenses/by/4.0/). Copyright 2019, MDPI.

Ran et al. fabricated electrochemical sensor using bromocresol green and Fe_3_O_4_ embedded in chitosan matrix for sensing serotonin, with a linear concentration range of 0.5–100 mM with lower limit of detection 80 nM [57]. In light of this imperative, Cai et al. have unveiled the morphology–dependent electrochemical sensing properties of iron oxide–graphene oxide nanohybrids for dopamine and uric acid (Fig. 9) [58]. Leveraging a facile meta-ion mediated hydrothermal method, the research yielded distinct morphologies of iron oxide nanoparticles (Fe_2_O_3_ NPs) encompassing cubic, rhombic, and discal configurations. In a quest to elevate electrochemical sensing prowess, the research team harnessed the exceptional electrocatalytic activity of discal Fe_2_O_3_ NPs (d- Fe_2_O_3_), coupling them with graphene oxide (GO) nanosheets. The synergistic interplay between discal Fe_2_O_3_ NPs and GO engendered remarkable electrocatalytic efficiency in the oxidation of dopamine (DA) and uric acid (UA). Significantly, this collaboration facilitated linear electrochemical responses for both DA and UA within concentration ranges of 0.02–10 μM and 10–100 μM, respectively. Impressively low limits of detection (LOD), specifically 3.2 nM for DA and 2.5 nM for UA, further underscored the sensitivity of the approach. Notably, the d- Fe_2_O_3_/GO nanohybrids showcased commendable selectivity and reproducibility, offering promising avenues for advanced electrochemical sensing applications.

#### 2.1.4 Manganese oxide-based biosensor

The utilization of manganese oxide in biosensing applications has gained substantial attention due to its unique physicochemical properties and potential for interfacing with biological systems. Manganese oxide, a transition metal oxide, possesses a diverse range of oxidation states, allowing it to facilitate redox reactions and electron transfer processes relevant to biosensing mechanisms. This distinctive property has led to the exploration of manganese oxide -based biosensors across various fields. Manganese oxide exhibits remarkable catalytic activity, making it an excellent candidate for electrochemical biosensors. Its inherent ability to mediate electron transfer between biomolecules and electrode surfaces has paved the way for the development of sensitive and efficient biosensing platforms. The tunable electrocatalytic behavior of manganese oxide, coupled with its compatibility with various biomolecules, holds promise for the detection of a wide array of analytes. Furthermore, manganese oxide nanostructures, including nanoparticles, nanowires, and nanosheets, offer high surface area-to-volume ratios, enhancing the immobilization of biomolecules and enabling signal amplification. These nanostructured forms of manganese oxide have been integrated into biosensing devices to achieve enhanced sensitivity and improved detection limits.

In the context of enzyme-based biosensors, manganese oxide has demonstrated its ability to facilitate the direct electron transfer between enzymes and electrodes. This feature eliminates the need for additional redox mediators, simplifying the sensor design and enhancing its stability. Enzymes immobilized on manganese oxide surfaces retain their bioactivity, allowing for reliable and reproducible biosensing. The diverse applications of manganese oxide -based biosensors encompass the detection of various analytes, including glucose, hydrogen peroxide, heavy metals, and environmental pollutants.

The MnO, MnO_2_, and Mn_3_O_4_ are three different oxides of manganese which are extensively studied and opted successfully as an electrode substance used in different biosensors. These oxides are non-hazardous, ecofriendly, easily available with low synthesis cost. Virtue of their quite high energy density and activity in alkaline medium, they have emerged an optimum material for designing biosensor for different analytes [[59], [60], [61], [62], [63], [64]]. In addition to this, oxides of manganese have four different dimensions starting from zero dimensions (0-D) to 3-D as oxides of zinc. Table 3 highlights all the four dimensions along with their applications in biosensing field. In comparison to 0-D, 1-D nanostructures 3-D NPs have more surface area (outer as well as inner) providing more reaction sites.

**Table 3:** Different dimensions of MnO_2_ nanostructures used in electrochemical biosensors.

Dimensions	Improved Electrode	Sensing Techniques	Sample	Biochemicals	Detection Limit	References
0-D	MnO_2_ NSPs@ GNR composites	Electrochemical	Honey	Glucose	0.1––1.4 mM	[65]
	MnO_2_ NPs/Polythiophene composite on GCE	Electrochemical	Human Serum	Dopamine	0.04––9.0 μM	[66]
	MnO_2_ NPs/Ta Electrode	Cyclic Voltammetry and Amperometry	Milk	H_2_O_2_	1––2 μM	[67]
	MnO_2_ NSPs GNR/SPCE	Cyclic Voltammetry and Amperometry	Honey	Glucose	0.1––1.4 mM	[65]
1-D	MnO_2_ NTs/Ag@C shell nanocomposites	Electrochemical	Toothpaste	H_2_O_2_	0.5 μM–5.7 mM	[68]
	M13-E4 @MnO_2_ NWs	Electrochemical	Human Serum, Local Peach Juice	Glucose	5 μM–2 mM	[69]
	Au/MnO_2_ NNDs/SPCE	Amperometry	Blood Plasma	Histamine	0.3––5.1 μM	[70]
	MnO_2_ NRs HBCs Nanocomposites/SPE	Cyclic Voltammetry and Chrono amperometry	Blood Sample	Glucose	28––93 μg/mL	[71]
2-D	MnO_2_ NSs/GCE	Electrochemical	SP2/0 Cells	H_2_O_2_	2––10 μM	[72]
	Lucigenin/MnO_2_ NSs/GCE	Electrochemiluminescence	Human Serum	Glutathione	10––2000 nM	[73]
	MWCNT-MnO_2_/rGO/Au electrode	Cyclic Voltammetry	Serum	Acetylcholine	0.1––100 μM	[74]
3-D	MnO_2_ NFs/3D-RGO @Ni foam	Electrochemical	Pork Sample	Ractopamine (RAC)	17––962 nM	[75]
	MNO_2_ NFs/N -rGO	Electrochemical	Human Serum	Dopamine	6––100 μM	[76]
	3D-MnO_2_ nanofibrous-mesh @GCE	Electrochemical	Blood and Urine Samples	Ascorbic Acid	0.20––10 mM	[77]

### 2.2 Quantum dot-based biosensors

Quantum dots (QDs) are semiconducting nanocrystalline materials with the diameter usually ranging from 2.0 nm to 10.0 nm [78]. Depending upon the size, these nanomaterials exhibit different colors viz.; QDs of diameter 5.0–6.0 nm give orange or red color while smaller QDs of diameter 2.0–3.0 nm are blue and green in appearance. The properties of QDs mainly depends on their size, shape, and structures. One of the main strategies for the synthesis of QDs is a top-down method in which large-sized carbon materials, such as graphite, graphene oxide, carbon nanotubes, carbon fibers extracted from different sources are broken down into small nano-sized quantum dots. They have been extensively employed as a substitute as the mimic fluorophores, for fabricating optical biosensors to detect organic compounds along with macromolecules [79]. Table 4 highlights the different types of QDs based biosensor used for detecting various analytes.

**Table 4:** The different types of QDs based biosensor.

S. No.	Type of QDs	Sensor type	Real sample	Analyte	Linear Range	References
1.	Ni-doped CdTe	Fluorescence	Plasma samples	Pyrazinamide	2–100 μM	[80]
2.	CdTe	Fluorescence	Biological fluids	Dopamine	0.5–10 μM	[81]
3.	MoS_2_/CdTe	Fluorescence	Milk samples	Tetracycline	0.1–1 μM	[82]
4.	CdS@MOF	Electrochemiluminescence	Human serum	Carcinoembryonic antigen	–	[83]
5.	CdTeS @SiO_2_	ImageJ software	Serum samples	Folic acid	5–80 μM	[84]
6.	α-FeOOH@ CdS/Ag	Electrochemiluminescence	–	17β-estradiol	0.01–10 pg mL^−1^	[85]
7.	ZnCdS QDs@MIP	Fluorescence	Vitamin C tablets	Ascorbic acid	1–500 μM	[86]
8.	MoS_2_/GQD	Electrochemical	Red wine samples	Caffeic acid	0.38–100 μM	[87]
9.	CdTe	Photoinduced electron transfer	Synthetic samples	Double-Stranded DNA	0.0874 μg mL^−1^ 20 μg mL^−1^	[88]
10.	Polymer CdTe/CdS	Fluorescence	Human body fluids	Glucose	0.2–5 mM	[89]

In 2013, Zhang et al. used nitrogen-doped carbon quantum dots (N-CQDs) for an effectual detection of mercury (II) ions with a lower detection limit of 0.23 μM [90]. In 2017, Saini et al. demonstrated a thiol functionalized fluorescent CQDs chemo sensor for arsenite detection, with a wide detection range of 5–100 ppb [91]. In 2017, Amjadi et al. demonstrated chemiluminescence sensor for the determination of indomethacin based on sulfur and nitrogen co doped CQDs, with a limit of detection of 65 μg L^−1^, and concentration range of 0.1–1.5 mg L^−1^ [92]. In the year 2017, Wang et al. used GQDs for designing a photoelectrochemical apta-biosensor for zeatin detection, with broad range [93].

**Fig. 10:**
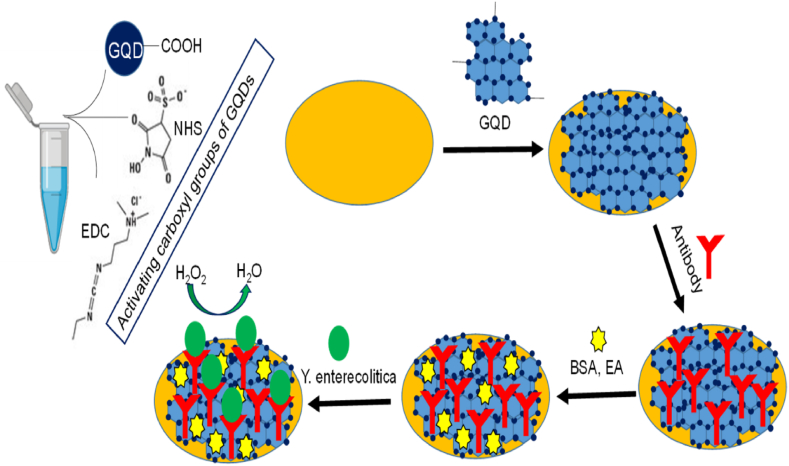
Graphene quantum dots (GQDs)-based immunosensor for Y. enterocolitica detection. Reproduced with permission from Ref. [94], with the permission of the Creative Commons Attribution 4.0 International License (http://creativecommons.org/licenses/by/4.0/). Copyright 2019, MDPI.

Yersinia enterocolitica, a gram-negative bacillus with its distinct rod-shaped morphology, is the causative agent of yersiniosis, a significant zoonotic ailment. This infection manifests through clinical symptoms such as mesenteric adenitis, acute diarrhea, terminal ileitis, and pseudoappendicitis, necessitating a robust detection strategy. To address this, an innovative approach was introduced by Sumeyra Savas and Zeynep Altontas in 2019, wherein an electrochemical sensor employing graphene quantum dots (GQDs) as nanozymes was meticulously developed and reported in their seminal work (Fig. 10) [94].

The study meticulously optimized the conditions for the assay, ensuring precision and accuracy in the detection process. This pioneering immunosensor, enriched with GQDs, was meticulously designed to specifically target Yersinia enterocolitica. The sensor showcased a remarkable ability to quantify the bacterium across a broad concentration range with extraordinary sensitivity. Notably, the limit of detection (LOD) was found to be impressively low, with LOD values of 5 cfu mL^−1^ for milk samples and 30 cfu mL^−1^ for serum samples. The system also exhibited remarkable specificity, further underscoring its reliability for targeted pathogen detection.

This novel electrochemical approach transcends its application to the detection of Yersinia enterocolitica, displaying the potential to revolutionize clinical and food sample analysis. With the elimination of pre-sample treatment, this methodology presents an efficient, swift, and cost-effective means to detect various pathogenic bacteria in diverse sample matrices. Thus, the innovative GQD-immunosensor holds the promise to transform the landscape of diagnostic and detection strategies for infectious agents, benefitting both clinical and food safety applications.

### 2.3 Nanowire-based biosensors

NWs are the solid wire like structures with nanometer diameters synthesized from semiconducting metal oxides, carbon and metal nanotubes. Virtue of their size, nanowire shows excellent mechanical, thermal, chemical, optical and electronic properties which are not seen in bulk materials. They are been highly exploited for the synthesis of biosensors with enhanced sensing/detecting limits [95,96]. Table 5 highlights some different metal NWs-based biosensors used for detecting different analytes.

**Table 5:** Different Metal NWs based biosensors.

Type of Sensor	Methods of Synthesis	Mechanism Used	Targeted Molecule	Range Limit	References
Pd@Ag Hollow NWs	LPNE/GRR	Chemiresistive	H_2_	900–100 ppm (100 ppm)	[97]
PAN@Pd yarn	Electrospinning	Chemiresistive	H_2_	4––0.0001% (1 ppm)	[98]
Pt NW PtOx NW	Electron beam lithography	Chemiresistive	H_2_	1000––0.5 ppm (100 ppm)	[99]
CoS_2_ NWs with Au NPs	Hydrothermal	Optical (Chemiluminescence)	H_2_O_2_	100–1 μM (0.03 μM)	[100]
Au NWs with DNA zyme	CVT	Optical (SERS)	UO_2_^2+^	10^−7^–10^−12^ M (1 pM)	[101]
PtNi jagged NWs	Solvothermal	Electrochemical	Caffeic Acid	0.75––600 μM (0.05 μM)	[102]
Cu_3_P NWs	Hydrothermal	Electrochemical	Glucose	1––0.005 mM (0.32 μM)	[103]
Ni/Au Multilayer NWs	Electrodeposition	Electrochemical	Glucose	2––0.0025 mM (0.1 μM)	[104]
G/Au NWs	Hydrothermal	Electrochemical (Cyclic Voltammetry)	Tulobuterol	7.6––0.076 μmolL^−1^ (0.0136 μmolL^−1^)	[105]
Ag NWs	Commercial	Piezoresistive	Strain	80–0% Strain (0.2%)	[106]
Au NWs	Oriented attachment	Chemiresistive	DNA	1––0.001 nM (1 pM)	[107]
Au NWs	Nanoimprint lithography	Electrochemical (SWV)	C-reactive protein	220–5 fg mL^−1^ (2.25 fg mL^−1^)	[108]
Ni NWs	Electrodeposition	Electrochemical (Cyclic Voltammetry)	HCHO	20––0.01 mM (0.8 μM)	[109]
AuPt NWs network with PDa Coating	Hydrothermal	Electrochemical	Pesticide	1000––0.5 ng/L (0.185 ng/L)	[110]

Further, in the year 2012, Hakim et al. fabricated a poly-silicon NWs biosensor for sensing the joining capacity of two inflammatory biomarkers with wide range of concentration and good detection sensitivity [111]. In 2017, Irrera et al. demonstrated label-free optical silicon Nws-based biosensors to detect the C-reactive protein in human serum, with the detection range of 10^−2^ μg/mL to 100 μg/ml [112]. In year 2018, Priolo et al. prepared and used silicon nanowires optical biosensors for ultrasensitive genome detection extracted from human blood [113].

**Fig. 11:**
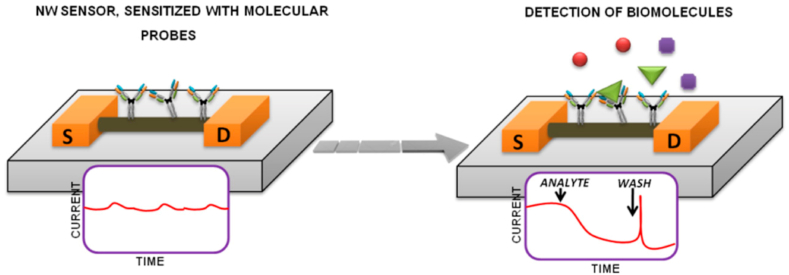
The schematic illustration for *Si*-NW-sensors to detect biomolecules. Reproduced with permission from Ref. [114], with the permission of the Creative Commons Attribution 4.0 International License (http://creativecommons.org/licenses/by/4.0/). Copyright 2021, MDPI.

In the year 2021, Ivanov and colleagues embarked on a pivotal scientific endeavor that concentrated on the exploration of cancer-associated genetic markers through the utilization of silicon nanowire field-effect transistors (Si-NW FETs) [114]. This investigation was strategically designed to capitalize on the inherent advantages of *Si*-NW FETs, particularly their compatibility with established and widely utilized mass production technologies. This pursuit of optimizing and integrating state-of-the-art technologies has significant implications for the advancement of diagnostics and detection in cancer research, as exemplified in Fig. 11.

Central to their study was the intricate scheme of *Si*-NW sensors, which serves as a foundation for the nanowire-based detection of biomolecules, specifically cancer-related genetic markers. The *Si*-NW FET configuration is envisaged to offer exceptional sensitivity and precision in the identification of these markers, underpinned by the unique electrical properties of nanowires. The miniaturized dimensions and enhanced surface-to-volume ratio of silicon nanowires inherently enable the detection of molecular interactions at a remarkably intricate level. By capitalizing on these inherent attributes, the researchers aimed to establish a robust and reliable methodology for detecting cancer-specific biomolecular signals.

### 2.4 Nanorods-based biosensors

NRs as the name suggest are the rods having dimensions range from 1 to 100 nm is synthesized chemically from different materials such as graphene, graphene oxide, oxides of various metals and other semiconducting materials [115,116]. These NRs have shown excellent potential in the field of biosensing for the detection of nucleic acids, different carbohydrates, metal ions etc.

In the year 2013, Sun et al. used graphene NRs and graphene oxide to prepare a biosensor to detect bovine IgG [117]. Later in the year 2017, Hahn et al. designed afield effect transistor (FET) biosensor using zinc oxide NRs for the detection of phosphate [118]. Further Zhu et al. in 2018used the same FET biosensor for glucose monitoring with high sensitivity and concentration detection limit of 1 μM [119]. Liu et al., in 2019 created a fluorescence resonance energy transfer biosensor for sensing lead ions using gold NRs and carbon dots [120]. Bagyalakshmi et al. in year 2020 fabricated a ZnO NRs-based enzymatic glucose biosensor on a chitosan film, with linear range of glucose concentrations from 10 μM to 40 μM [121].

**Fig. 12:**
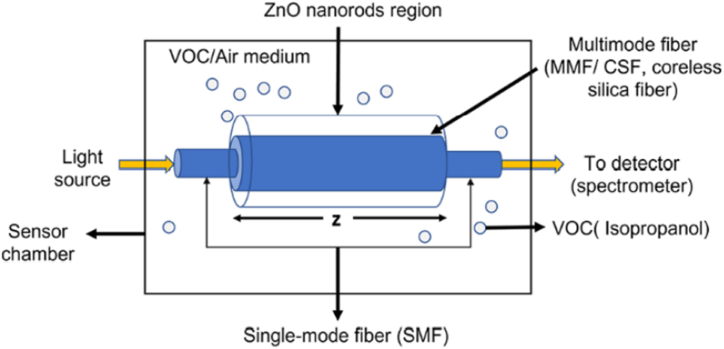
ZnO nanorods coated optical fiber sensor for volatile organic compounds (VOC) biomarker detection. Reproduced with permission from Ref. [122], with the permission of the Creative Commons Attribution 4.0 International License (http://creativecommons.org/licenses/by/4.0/). Copyright 2022, MDPI.

Volatile organic compounds (VOCs) are ubiquitous in the environment, often existing as gaseous species under specific temperature and pressure conditions. These compounds emanate from a diverse array of sources, including household products, paints, fuels, personal care items, waxes, and industrial processes, and become integral components of the atmosphere. The quantification of VOC concentrations in exhaled breath has garnered considerable attention due to their potential as indicative biomarkers for various chronic diseases. Notably, acetone and isopropanol have emerged as significant biomarkers for type 1 diabetes and lung cancer, respectively, emphasizing the clinical relevance of VOC analysis. In a recent groundbreaking study by Kankan Swargiary et al. an innovative optical fiber sensor employing zinc oxide (ZnO) coating was introduced for the selective detection of a volatile organic compound (VOC) biomarker associated with diabetes, specifically targeting isopropanol (IPA) markers [122]. The sensor configuration incorporated a coreless silica fiber (CSF) bridging two single-mode fibers (SMFs), forming a structured SMF–CSF–SMF architecture (depicted in Fig. 12). The CSF region functioned as the sensing zone, harnessing multimode interference (MMI) to intensify light interaction at the interface between the fiber and the sensing medium, thereby enhancing sensitivity levels. Numerical simulations were meticulously employed to optimize the CSF length, ensuring maximal coupling efficiency at the output.

The surface of the CSF was ingeniously functionalized via a hydrothermal ZnO nanorod growth process, facilitated at low temperatures. This innovative step facilitated the establishment of a robust sensing platform without compromising the structural integrity of the fiber. The constructed optical fiber-based sensor was subjected to rigorous testing using various concentrations (20%, 40%, 60%, 80%, and 100%) of isopropanol (IPA). The sensor exhibited exceptional potential in accurately detecting isopropanol vapor, showcasing an impressive sensitivity of 0.053 nm/% IPA vapor. These findings collectively underscore the sensor's capacity to discriminate and quantify the presence of isopropanol, thereby showcasing its potential utility in non-invasive monitoring for diabetes-related applications and potentially extending its applications to broader medical contexts.

### 2.5 Carbon nanotubes-based biosensors

Carbon nanotubes (CNTs), also known as buckytubes were first reported by SsumioIjima in year 1991. They are hollow carbon structures having diameters in nanoscale. They display proper arrangement of carbon atoms linked via sp^2^ bonds [123], making them quite strong and stiff materials. They are most extensively explored class of nanomaterials for making biosensors applied for different diagnostics purposes in medical and other research areas and serve as a scaffold of immobilization of biomolecules at their surface. In the year 2006, Tang et al. prepared a single-walled carbon nanotube (SWNT) based DNA sensors with great sensibility and response [124]. In year 2013, Li et al. fabricated a biosensor using semiconducting single-wall carbon nanotubes (s-SWCNTs) to detect the dopamine, with a very low detection limit of 10^−18^ mol/L at room temperature [125]. There is a list of CNTs-based biosensors with different analytes as shown in Table 6.

**Table 6:** List of CNTs-based biosensors with different analytes.

Sensor Types	Type of Mechanism	Methods of Synthesis	Analyte	LOD and Range	References
CNTs	Field–effect transistor	OTS masking	Aquaporin-4	1 ng/L, 1–10^6^ ng/L	[126]
	Amperometric	Dielectrophoresis	Streptavidin	100 aM, 100––10^6^ aM	[127]
	Amperometric	Dielectrophoresis	HER2 Antibody	10 fM, 10–10^5^ fM	[127]
	Chemiresistive	Direct Contact Printing	H5N1 DNA Sequence	SWCNT- 2pM, 2––200 pM; MWCNT- 20 pM, 20––2000 pM	[128]
	Chemiresistive	Drop–Coat	Prostate-Specific antigen	1.18 ng/mL, 0––1000 ng/mL	[129]
	Field–effect transistor	Dielectrophoresis	Cortisol	50 nM, 50––1000 nM	[130]
CNTs	Field–effect transistor	Dielectrophoresis	NPY	500 pM, 500–10^6^ pM,	[130]
	Field–effect transistor	Dielectrophoresis	DHEAS	10 nM, 10––1000 nM	[130]
	Field–effect transistor	OTS masking	Aspergillus Niger	N/A	[131]
	Field–effect transistor	Immersed in CNTs solution	DNA	60 aM, 100––1000 aM	[132]
	Field–effect transistor	Immersed in CNTs solution	Micro vesicle	6 particles per mL, 6––6 × 10^6^ particles per mL	[132]
	Field–effect transistor	Catalytic Chemical vapor deposition	N_2_^+^ ion	Single ion	[133]
CNTs	Amperometric	Drop–Coat (Paper Filter)	Formaldehyde	0.016 ppm, 0.05––6.7 ppm	[134]
	Chemiresistive	Dielectrophoresis	H_2_	10 ppm, 10 ppm–4%	[135]
	Chemiresistive	Dielectrophoresis	NO_2_	0.5––20 ppm	[136]
	Chemiresistive	Immersed in CNTs solution	H_2_	0.89 ppm	[137]
	Chemiresistive	Drop–Coat	NH_3_	100 ppb, 1.5––20 ppm	[138]
	Chemiresistive	Drop–Coat	N-nitroso dialkylamine	1 ppb, 0––1000 ppb	[139]
	Chemiresistive	Spray deposition	NH_3_	10 ppm, 10––100 ppm	[140]
	Chemiresistive	Spray deposition	CO_2_	600 ppm, 600––7000 ppm	[140]
CNTs	Chemiresistive	Spray deposition	CO	3 ppm, 3––27 ppm	[140]
	Chemiresistive	Spray deposition	Ethanol	17 ppm, 17––70 ppm	[140]
	Chemiresistive	Dielectrophoresis	Tetrahydrocannabinol	0.163 ng, 0.0018–0.8262 μg	[141]
	Chemiresistive	Drop–Coat	NH_3_	2 ppm, 2––40 ppm	[142]
	Chemiresistive	Drop–Coat	NO_2_	2 ppm, 2––40 ppm	[142]
	Chemiresistive	Catalytic Chemical vapor deposition	Toluene	50 ppm, 50––500 ppm	[143]
	Spin-coat	Field–effect transistor	DNA (cDNA from Cancer Cell)	880 ng/L, 50––5 × 10^6^ pM	[144]

**Abbreviations**: LOD = Limit of detection, CNT = Carbon Nanotubes, HER2 = Human epidermal growth factor receptor 2, DHEAS = Dehydroepiandrosterone sulfate.

Neurotransmitters play a fundamental role in orchestrating crucial physiological functions within the human body, particularly in mediating intricate chemical communications within neuronal networks of the brain. However, a comprehensive understanding of their intricate mechanisms remains largely uncharted territory, primarily due to the scarcity of effective tools capable of capturing their concentration dynamics with spatiotemporal precision. Over the last few decades, significant strides have been made in devising analytical methodologies aimed at quantifying neurotransmitter levels.

Janssen et al. prepared CNTs-based biosensor for detecting bovine serum albumin (BSA), with excellent detection limit [145,146].

**Fig. 13:**
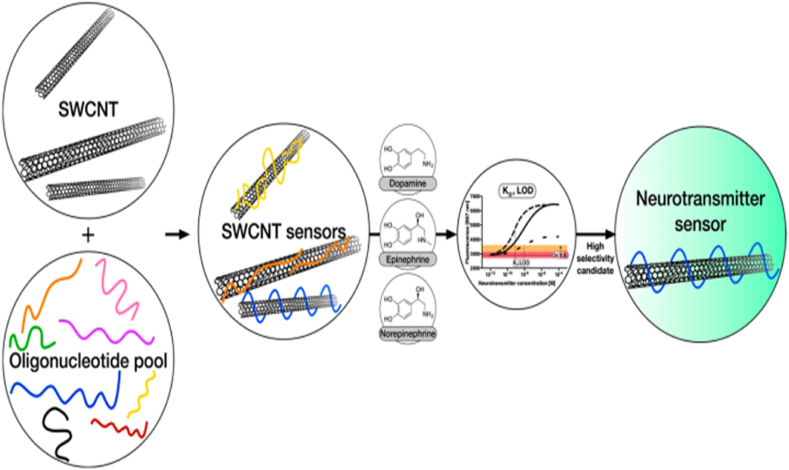
Fluorescent carbon nanotube-based neurotransmitter sensors. Reproduced with permission from Ref. [147], with the permission of the Creative Commons Attribution 4.0 International License (http://creativecommons.org/licenses/by/4.0/). Copyright 2017, MDPI.

In an another notable contribution to this field, Florian et al. presented an innovative approach encompassing the development and thorough characterization of fluorescent carbon nanotube-based sensors dedicated to neurotransmitter detection [147]. In their study, Florian and colleagues employed a systematic manipulation of the organic phase surrounding single-walled carbon nanotubes (SWCNTs) to engineer a spectrum of sensors, each endowed with distinct selectivity and sensitivity profiles tailored for catecholamine neurotransmitters (Fig. 13). The investigation yielded a comprehensive understanding of the sensors' performance, establishing a nuanced interplay between the DNA sequences and the SWCNT platform. Of particular significance is the sensors' capacity to distinguish between diverse catecholamine neurotransmitters or detect them amidst the presence of structurally similar interfering compounds. This remarkable capability addresses a critical limitation in existing methodologies, allowing for more accurate and nuanced measurements.

The implications of this research are noteworthy, as DNA-functionalized SWCNT-based sensors exhibit the potential to revolutionize our capacity to delve into neurotransmitter signaling within complex biological milieus. By virtue of their heightened selectivity and sensitivity, these sensors hold promise as invaluable tools in deciphering the intricate interplay of neurotransmitters in both health and disease contexts, thereby offering a stepping stone toward unraveling the complexities of neurological processes at unprecedented levels of detail.

### 2.6 Dendrimer-based biosensors

**Fig. 14:**
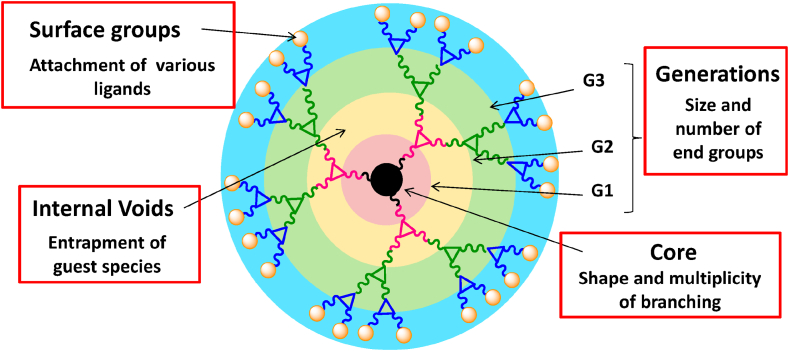
Different elements of Dendrimers. Reproduced with permission from Ref. [148], with the permission of the Creative Commons Attribution 4.0 International License (http://creativecommons.org/licenses/by/4.0/). Copyright 2015, MDPI.

In recent years, dendrimers have garnered significant attention as versatile nanoscale architectures with promising applications in the field of biosensors. Dendrimers, three-dimensional hyperbranched macromolecules, offer a unique combination of properties, including well-defined structures, tunable surface functionalities, and high branching densities (Fig. 14). These attributes make them well-suited for engineering biosensing platforms with enhanced sensitivity, selectivity, and stability.

The incorporation of dendrimers into biosensor design leverages their multifunctional nature. Dendrimers can serve as molecular scaffolds for immobilizing biomolecules such as enzymes, antibodies, and nucleic acids. This controlled immobilization not only maintains the bioactivity of these recognition elements but also facilitates their precise arrangement, leading to improved interactions with target analytes.

Moreover, dendrimers possess intrinsic signal amplification capabilities owing to their high surface area and numerous functional groups. This unique feature enables the immobilization of multiple reporter molecules or signal tags, resulting in an amplified response upon target binding. This enhanced signal output enhances the biosensor's detection limit and dynamic range, rendering it more suitable for accurately quantifying low concentrations of analytes.

Dendrimer-based biosensors find applications across diverse domains, including medical diagnostics, environmental monitoring, and food safety. For instance, in medical diagnostics, dendrimer-enhanced biosensors have shown promise in detecting specific biomarkers associated with diseases such as cancer, diabetes, and infectious disorders. Their tunable surface chemistry allows for tailoring sensor surfaces to interact with distinct target molecules, enabling the development of highly specific assays. Furthermore, dendrimer-modified electrodes have demonstrated improved electron transfer kinetics, enhancing the overall sensor performance in terms of response time and stability. This attribute is especially advantageous in electrochemical biosensors, where rapid and accurate measurements are paramount.

The remarkable potential of dendrimer-based biosensors lies in their ability to bridge the gap between nanotechnology and biorecognition elements. By synergistically combining the unique attributes of dendrimers with the specificity of biomolecular recognition, these biosensors hold the promise of advancing the boundaries of biosensing technology. As research in this field continues to evolve, it is anticipated that dendrimer-enhanced biosensors will play an increasingly pivotal role in addressing pressing challenges in healthcare, environmental monitoring, and beyond.

The extensive branching in the dendrimers provides immense outside area for binding of different biologically active molecules. They are basically made up of three subunits viz.; the central core unit, the branching dendrones, and the outer surface ligands [149]. Tumor markers, esteemed for their diagnostic potential, offer invaluable insights into the intricacies of diverse tumors, aiding in prognosis and unraveling the molecular underpinnings of tumorigenesis. Within this context, the emergence of nanotechnology-driven approaches has ushered in novel avenues for detecting these markers, promising heightened accuracy and clinical utility.

In a recent contribution, Ushna Laraib orchestrates a comprehensive literature review that accentuates the pivotal role of nanomaterials in the realm of tumor biomarker detection [150]. Notably, the review encapsulates an array of prominent tumor markers, including prostate-specific antigen (PSA), human carcinoembryonic antigen (CEA), alpha-fetoprotein (AFP), human chorionic gonadotropin (hCG), human epidermal growth factor receptor-2 (HER2), cancer antigen 125 (CA125), cancer antigen 15-3 (CA15-3, MUC1), and cancer antigen 19-9 (CA19-9).

The landscape of theranostics is undergoing a paradigm shift with the advent of RNA aptamers, emerging as a potent arsenal against a myriad of disorders. Leveraging their remarkable structural flexibility, RNA aptamers demonstrate a unique capability to intricately fold and engage with a diverse array of nanostructures, macromolecules, cells, and viruses. This multifaceted potential has propelled them to the forefront of cutting-edge theranostic research.

In a recent scholarly endeavor, Mahtab and colleagues have orchestrated a comprehensive literature review, unveiling a treasure trove of insights into the evolutionary landscape of RNA aptamers [151]. With a meticulous exploration, the review delves into the intricate facets of their development, classification, nanomerization, and strategic modification. Notably, it proffers an incisive account of their applications within the realm of cancer theranostics, signifying a substantial leap forward in precision medicine.

Dendrimers have been extensively used for designing numerous biosensors based on electrochemical, fluorescence and impedance methods applied for different diagnostic purposes. This sensor possesses high analytical sensitivity, stability, and reproducibility [152,153]. In 2016, Ou et al. fabricated electrochemiluminescence (ECL) biosensor using Ag nanocubes–polyamidoamine dendrimer–luminol–glucose oxidase (AgNCs–PAMAM–luminol–GOx) to detect the concanavalin A (Con A), with two wide linear response ranges from 0.005 to 0.1 ng/mL and 0.1–20 ng/mL [154]. In the year 2017, Dervisevic et al. fabricated a novel electrochemical urea biosensor based on ferrocene poly(amidoamine) (Fc-PAMAM) dendrimers combined with multi walled carbon nanotubes (MWCNTs), with detection limit of 0.05 mM, and sensitivity of 1.085 μA/cm^2^/mM [155]. Further in the year 2019, Baker et al. used polyamidoamine (PAMAM) dendrimer sensor for the detection of dengue fever [156]. To conclude the application of various nanomaterials used for biosensor development, Table 7 was prepared so that the reader can get proper idea of the work carried out in last two decades.

**Table 7:** List of various nanomaterials used in biosensor development over the last two decades.

Nanomaterial	Transducer	Analyte	Detection Limit	Linear Range	Ref.
Au NBPs	SPR Impedimetric	Aflatoxin B1(AFB1) Aflatoxin B1 (AFB1)	0.4 nM 0.1 nM	0.1––500 nM 0.1––25 nM	[157]
Au NPs	Electrochemical	Uranyl	0.3 μg L^−1^	2.4––480 μg L^−1^	[158]
Au NPs	Fluorescent	Pb^2+^	16.7 nm	50 nm–4 μm	[159]
Au/CdS QDs/TNTs	Electrochemical Electrochemical	Cholesterol H_2_O_2_	0.012 μM 0.06 μM	0.024––1.2 mM 18.73––355.87 μm	[160]
Au NPs	Electrochemical	*E. coli*	15 CFU mL^−1^	10––106 CFU mL^−1^	[161]
Au NP-MoS_2_ -rGO	SAW	Carcinoembryonic antigen (CEA)	0.084 ngmL^−1^	36.58 ng mL^−1^	[162]
Au/rGO	Electrochemical	miENA-122	1.73 pM	10 μm–10 p.m.	[163]
Au NPs/TiO_2_	Electrochemical	H_2_O_2_	5 μm	65––1600 μm	[164]
Ag NPs	Colorimetric	H_2_O_2_ Glucose Fe^2+^	0.032 μm 0.29 μm 0.54 μm	0.05––7.5 μm 1.5––3.0 μm 1––90 μm	[165]
Ag/Pd NPs	Electrochemical	Ractopamine Clenbuterol Salbutamol	1.52 pg mL^−1^ 1.44 pg mL^−1^ 1.38 pg mL^−1^	0.01––100 ng mL^−1^ 0.01––100 ng mL^−1^ 0.01––100 ng mL^−1^	[166]
Ag@CQDs-rGO	Electrochemical	Dopamine	0.59 nm	0.1––300 μm	[167]
Ag NP-MWNT	Electrochemical	Glucose	0.01 mM	0.025––1.0 mM	[168]
Ag NPs	Electrochemilumi-nescence	Mucin 1	0.37 fg mL^−1^	1.135 fg mL^−1^ -0.1135 ng mL^−1^	[169]
Pt NPs	Voltammetric	Adrenaline	2.93 × 10^−4^ mol L^−1^	9.99 × 10^−1^–2.13 × 10^−4^ mol L^−1^	[170]
Pt NPs/RGO–CS–Fc	Electrochemical	H_2_O_2_	20 nm	2.0 × 10^−8^ M − –3.0 × 10^−8^ M	[171]
Pt–Fe_3_O_4_@C	Amperometric	Sarcosine	0.43 μm	0.5––60 μm	[172]
Pt NFs/PANi	Cyclic Voltammetry	Urea	10 μm	20 mM	[173]
Pt@CeO_2_ NM	Electrochemical	Dopamine	0.71 nM	2––180 nM	[174]
Pd/Co-NCNT	Electrochemical	Hydrazine	0.007 μm	0.05––406.045 μm	[175]
Pd/CNF/[M3O A]^+^[NTF2]^−^		H_2_	0.33 nM	1.00––35.0 nM	[176]
Cu NPs/Rutin/MWCNTs/IL/Chit/GCE	Cyclic Voltammetry	H_2_O_2_	0.11 μm	0.35––2500 μM	[177]
Cu/rGO-BP	Electrochemical	Glucose	11 μm	0.1––2 mM	[178]
Cu_2_O@CeO_2_–Au	Amperometric	PSA	0.0001––100.0 ng mL^−1^	0.03 pg mL^−1^	[179]
Ni/Cu MOF	FET	Glucose	0.51 μM	1 μM–20 mM	[180]
NiO/PANINS	Amperometric	Glucose	0.06 μM	1––3000 μM	[181]
NiO@Au	Electrochemical	Lactic acid	11.6 μM	100.0 μM–0.5 M	[182]
Co_3_O_4_ NCs	Electrochemical chip	Glutamate	10 μM	10––600 μM	[183]
Co_3_O_4_–Au	Photoelectricalche-mical	miRNA-141	0.2 pM	1 pM–50 nM	[184]
MnO–Mn_3_O_4_ @rGO	Impedimetric	H_2_O_2_	0.1 μM	0.004––17 mM	[185]
MnO_2_ NFs	Impedimetric	Salmonella	19 CFU mL^−1^	3.0 × 10^1^––3.0 × 10^6^	[186]
Fe_2_O_3_/NiO/Mn_2_O_3_ NPs	Electrochemical	Folic acid	96.89 ± 4.85 pM	0.1 nM–0.01 mM	[187]
ZnO-rGO	Cyclic Voltammetric	Dopamine	8.75 ± 0.64pM	0.1––1500 pM	[188]
ZnO NRs	FET	Phosphate	0.5 mM	0.1 μM–7.0 mM	[189]
ZnO NFs	Optical	Amyloid	2.76 μg	2––20 μL	[190]
Ca/Al–ZnO NPs	Semiconductor	CO_2_	200 ppm	0.25––5 RH%	[191]
Cr doped SnO_2_ NPs	Voltammetric	Riboflavin	107 nM	0.2 × 10^−6^––1.0 × 10^−4^ M	[192]
TiO_2_/APTES	Impedimetric	Glucose	24 μmol	50––1000 μmol	[193]
TiO_2_ NTs	Photoelectrochemical	Asulam	4.1 pg mL^−1^	0.02––2.0 ng mL^−1^	[194]
MoO_3_@RGO	Electrochemical	Breast cancer	0.001 ng mL^−1^	0.001––500 ngmL^−1^	[195]
Graphene QDs	Electrochemical	Cu^2+^	1.34 nM	0.015––8.775 μM	[196]
Graphene QDs	Fluorescence	Lung cancer^+^	0.09 pg mL^−1^	0.1 pg mL^−1^–1000 ng mL^−1^	[197]
CdTe/CdS//ZnS core/shell /shell QDs	Fluorescence	l-ascorbic acid	1.8 × 10^−9^ M	8.0 × 10^−9^––1.0 × 10^−7^ M	[198]
NSETamptamer@Fe_3_O_4_@GOD and MoS_2_	Magnetic fluorescence	Tumor cell (EpCAM)	1.19 nM	2––64 nM	[199]
Au NPs@PDA @CuInZnSQDs	Electrochemiluminescence	P53 gene	0.03 nmol L^−1^	0.1––15 nmol L^−1^	[200]
CaM/SiNW FETs	FET	Protein	7 nM	10^−8^–10^−6^ M	[201]
Si NWs	FET	Dengue virus	2.0 fM	1 μM–10 fM	[202]
ZnO NRs	FET	Phosphate	0.5 mM	0.1 μM–7.0 mM	[203]
G/Au NR/PT	Electrochemical	HPV DNA	4.03 × 10^−14^ m L^−1^	1.0 × 10^−13^––1.0 × 10^−10^ m L^−1^	[204]
Graphene-Au NRs	Amperometric Voltammetric	NADHEthanol	6 μM 1.5 μM	20––160 μM 5––377 μM	[205]
LAC-CNTs-SPCE	Electrochemical	Para-cresol	0.05 ppm	0.2––25 ppm	[206]
Co_3_O_4_-CNT/TiO_2_	Photoelectrochemical	Glucose	0.16 μM	0––4 mM	[207]
CNT thin-film transistor (TFT)	Thin film transistor (TFT)	DNA	0.88 μg L^−1^	1.6 × 10^−4^–5 μmol L^−1^	[208]
GQDs-MWCNTs	Electrochemical	Dopamine	0.87 nM	0.005––100.0 μM	[209]
CNT/Au NPs	Amperometric	Choline	15 μM	0.05––0.8 mM	[210]
PAMAM dendrimer	Optical fiber	DENV 2E	19.53 nm nM^−1^	0.1 pM–1 μM	[211]
SAM/NH_2_rGO/PAMAM	SPR	DENV 2E	0.08 pM	0.08 pM––0.5 pM	[212]

## 3 Challenges and emerging trends in nanobiosensors

The impending global population projection of 8.5 billion by 2030 brings forth significant challenges to the healthcare infrastructure, including the availability of diagnostic resources, testing facilities, and affordable medical care. Such a scenario may lead to increased costs associated with diagnostic procedures, impacting healthcare accessibility, particularly in developing countries like India. The pressing need for immediate and portable diagnostics has spurred the development of Point-of-Care Technologies (POCT) that integrate cutting-edge technologies to provide rapid results [213,214].

The realm of nanotechnology has witnessed remarkable advancements, with nanomaterials like quantum dots, graphene, carbon nanotubes, and nanocomposites prominently harnessed for diagnostic applications. While nanobiosensors initially made their debut in glucose detection [215], several challenges have surfaced in bringing nanoparticle-based biosensors to the commercial market. Critical challenges include addressing public and regulatory concerns surrounding safety, ethical considerations, and the establishment of universal standards for assessing nanobiosensor safety.

The escalating demand for POCT has extended to various biological sample analyses, encompassing blood, urine, saliva, and DNA, and even encompassing environmental pollution monitoring, biochemical testing, and pathogen detection. The integration of artificial intelligence, cyber-physical systems, and cutting-edge technologies has propelled the intelligent nanobiosensors market [216]. However, the multidisciplinary nature of nanobiosensors calls for advances in sciences, electronics, and mechanical design to enhance sensitivity and selectivity for applications spanning in vitro diagnostics, pharmaceuticals, drug delivery, and pathogen detection [217].

Furthermore, nanobiosensors offer substantial promise to healthcare practitioners, researchers, and scientists by enabling precise detection of nucleic acid sequences, proteins, enzymes, and biomarkers associated with various conditions and diseases. Conventional assays, though available, often suffer from extended processing times, a need for multiple analytes, and the risk of erroneous outcomes. Consequently, there is a compelling demand for rapid, reliable, and cost-effective multiplexed screening capable of detecting diverse analytes.

Focusing on the fusion of nanoelectronics, sensors, and materials, the pursuit of eco-friendly nanobiosensors with applications in diverse fields like food analysis, environmental monitoring, and diagnostics has gained momentum. The evolution of diagnostic technologies remains pivotal, allowing healthcare professionals and researchers to glean accurate insights into disease pathways. To address these challenges and propel the nanobiosensor field forward, emphasis must be placed on pioneering nanomaterials and sensor technologies that efficiently bridge the gap between nanoscience and diagnostics, ultimately serving healthcare, environmental monitoring, and other industries requiring precision detection and monitoring [218].

## 4 Conclusions and future perspectives

Biosensors, a remarkable fusion of bioreceptors, transducers, and amplifiers, stand as versatile analytical tools capable of detecting a wide spectrum of analytes including heavy metal ions, carbohydrates, amino acids, gases, and disease-associated substances. This comprehensive review underscores the diverse types, classifications, and applications of biosensors. It particularly highlights the pervasive utilization and recent advancements in metal oxide nanoparticles (NPs), nanowires (NWs), nanorods (NRs), carbon nanotubes (CNTs), quantum dots (QDs), and dendrimers in designing NPs-based biosensors for a plethora of applications.

The adoption of these nanomaterials in biosensors capitalizes on their inherent attributes of heightened sensitivity, selectivity, reproducibility, and stability. The exceptional charge mobility, expansive surface area, and superior electrochemical characteristics of nanomaterials underpin their enhanced performance. As we cast a glance toward the future, the potential of nanobiosensors is boundless, with a trajectory marked by automation, integration, and miniaturization. The synergy of advanced technologies such as the Internet of Things (IoT), deep learning (DL), cloud computing, data analysis, cyber-physical systems (CPS), and artificial intelligence (AI) promises to drive their commercialization.

Nanobiosensors, borne from the convergence of nanotechnology, biotechnology, and sensor engineering, are primed to revolutionize Point-of-Care Testing (POCT). Looking ahead, these innovative devices hold immense promise in healthcare and beyond, offering real-time, on-site diagnostics and monitoring. In essence, the emergence of nanomaterials-based biosensors stands as a monumental achievement of our time, poised to reshape the landscape of diagnostics and catalyze transformative advancements in a wide array of domains.

## Author contribution statement

All authors listed have significantly contributed to the development and the writing of this article.

## Data availability statement

Data will be made available on request.

Declaration of interest's statement: The authors declare no conflict of interest.

## Declaration of competing interest

The authors declare that they have no known competing financial interests or personal relationships that could have appeared to influence the work reported in this paper.
